# Roger Sperry, the maverick brain scientist who was haunted by psyche

**DOI:** 10.3389/fnhum.2024.1392660

**Published:** 2024-04-11

**Authors:** Giovanni Berlucchi, Carlo Alberto Marzi

**Affiliations:** Department of Neurosciences, Biomedicine and Movement Sciences University of Verona, Verona, Italy

**Keywords:** neural bases of consciousness, connectionism vs. anticonnectionism, mind–body problem, brain hemispheric differences, split-brain

## Abstract

This paper describes the scientific figure of Roger Sperry as a maverick researcher, an original thinker who arrived at definitive notions about the working of the brain mostly by distancing himself from the prevalent views of his peers. After solving the riddle of the functions of the corpus callosum, he won a Nobel prize in physiology or medicine for identifying the different cognitive abilities of the disconnected right and left hemispheres of the human brain. He could have won another Nobel prize for his work on the prenatal formation of behavioral neuronal networks and their growth and development after birth. In the last part of his life, he fought a courageous but inconclusive battle for demonstrating that mental and spiritual factors can direct brain activity and behavior without violating the laws of orthodox neurophysiology. Some nodal points in his scientific career and some sources of inspirations for his thinking are identified and discussed within the historical background of the neurosciences of the twentieth century.

## The origins: two sources of inspiration for Sperry: Herrick, and Lashley

In 1938, when Roger Sperry with an M.A. in Psychology from Oberlin College had started to work for a doctorate under Paul Weiss at the University of Chicago, the University had recently lost two of its most important faculty members. One was Karl Lashley, Sperry’s future postdoctoral mentor, who had left Chicago in 1935 after being recruited from Harvard as “the best psychologist in the world” (Boring, 1952). The other was Charles Judson Herrick who had retired in 1937 after working for 30 years as professor of neurology in the Department of Anatomy and Psychology (Bartelmez, 1973; Kingsland, 1993). There was never a formal academic relation between Herrick and Sperry, but we will provide evidence that Herrick’s last book was influenced by Sperry’s original ideas on cerebral organization, while in turn Sperry’s mature reflections on brain science and spiritual values belong in their own right to the psychobiological tradition established by Herrick. Despite the name of his professorship, Herrick was not a medical man. A son of a Baptist preacher, he was a dropout from a Baptist clerical school who had then chosen a career in science and earned a doctorate in biology from Columbia University. As a professor of neurology at Chicago University, Herrick was very influential in widening the concept of neurology from a clinical specialty practiced by physicians to the broad biological academic subject which today we call neuroscience. For him neurologists were not only the medical specialists who treated patients affected by neurological or psychiatric disorders, but also all investigators, medical and non-medical, who pursued the understanding of the nervous system of humans and animals with various aims and techniques. After receiving his basic training in science from his older brother Clarence, an erudite naturalist who died young, Herrick had become a first-rate explorer of the fine morphology of the nervous system of many animal species, from fishes to amphibia to rats and humans, always seeking specific relations between neural structure and behavioral functions. The special interest of the two Herrick brothers in the comparative study of the nervous system is attested by their role in the history of the Journal of Comparative Neurology, founded by Clarence in 1891. Following Clarence’s early death, the journal was run for many decades by his younger brother who almost heroically overcame various discouraging difficulties, such that in 2024 the journal can boast to be the oldest basic neuroscience journal, continually published for 133 years. In addition to his excellent performance as a researcher and teacher of neural science, Herrick also successfully organized a Neurology Club of the University of Chicago, an interdisciplinary instructive and stimulating discussion group whose members included faculties and researchers of most biological departments as well as clinical neurologists and other clinicians. Lashley and Weiss, who had been trained as zoologists but then had turned their research interest to the nervous system, Weiss to neuroembryology and Lashley to the coalescence of neurology and psychology, were members of the club along with Percival Bailey and Paul Bucy (neurologists and neurosurgeons), Stephen Polyak and David Bodian (neuroanatomists), Heinrich Kluver (experimental psychologist), Roy Grinker (neuropathologist), Ralph Gerard, Nathaniel Kleitman, Ralph Lillie (neurophysiologists) and several others. Herrick maintained that psychology is a biological discipline, but against the tenets of strict behaviorism he argued that mental events and states are vital conditions that can have causal power in the production of behavior. He also argued that subjectivity can be studied scientifically with appropriate analyses of controlled introspective reports. On the initial inspiration of his brother Clarence, he employed the term psychobiology, already in use with multiple different meanings and connotations in psychology and psychiatry (Dewsbury, 1991), to refer to the coherent scientific integration of the seemingly disjointed objective and subjective domains of human experience. Both Herrick and Lashley considered themselves behaviorists and biological determinists, but their respective theoretical positions differed in different ways from those of the strict or radical behaviorists who denied the existence of consciousness. As already stated, for Herrick scientifically controlled introspection revealed that consciousness was an important factor in behavior and a real cause of conduct in humans and possibly, at least to some extent, also in other animals. Lashley instead was convinced that facts of conscious experience exist but are unsuited to any form of scientific treatment other than their complete reduction to chemical and physical events (Lashley, 1923). He was also very critical of the behavioristic explanation of learning based on the modification by use of fixed reflex pathways mediating responses to environmental stimuli. In addition, Herrick and Lashley disagreed deeply about the relations between science and humanism.

The historian of science Sharon Kingsland writes that in the struggle for psychobiology at the University of Chicago “Herrick represented a generation of active discipline builders who, in the process of expanding the authority of science, were trying to reconcile science and humanistic philosophy. For Lashley the philosophical quest was irrelevant: the scientific and humanistic modes of thought were antagonistic” (Kingsland, 1993). This marked theoretical disagreement did not prevent a fertile interaction between Herrick and Lashley in neurological discussions which led to their independent formulation of converging theories about the general organization of brain functioning. For another historian of science, Nadine Weidman, the Herrick-Lashley controversy was much more than a struggle at the University of Chicago. Her 1999 book “Constructing Scientific Psychology,” subtitled “Karl Lashley’s Mind-Brain Debates,” is written from a social constructivist perspective. In public Lashley expressed himself in favor of a complete independence of science from cultural, political, and spiritual factors, and claimed that his interpretation of the experimental results was objective, neutral, and above all value-free. But in his private correspondence he made no secret of his hereditarian, racist and misanthropic convictions. According to Weidman (1999), Lashley did his experiments on brain-lesioned rats only in controlled laboratory conditions to reduce the effects of environment and to enhance those of heredity on the experimental results. His final aim, she argues, was to show that intelligence was hereditary and not improvable by education, in support of his anti-progressive political views which countered racial desegregation and favored the maintenance of the social status quo. Contrary to Lashley, Herrick and his followers viewed science as an originator of knowledge with practical relevance to human society, as well as a source of humanistic values naturally leading to progress toward full democracy, peace, and prosperity. Lashley’s mass action and equipotentiality principles might apply to the brains of both rats and men, but “men are bigger and better than rats.” Herrick’s psychobiology was aimed at identifying the mechanisms proper of the human brain that allow humans to have beliefs, reasons and aims that rats’s brains cannot sustain. Weidman (1999) describes the conflict between the views of Herrick and those of Lashley as a clash between a humanitarian progressive psychobiology and an anti-progressive neuropsychology, where the use of the second term seems justified by the fact that since 1937, at Harvard, Lashley was the first person in history to bear the title of research professor of neuropsychology (Orbach, 1982). Based on Bruce (1985), Weidman was aware that Lashley neither invented the term nor made a frequent use of it, the meaning of which he intended solely as the study of the higher functions of the normal brain. That he was also not particularly fond of the term is attested by the following autobiographic reminiscence of Karl Pribram, a neurosurgeon who in the 1940s worked with Lashley and his collaborators at the Yerkes primate laboratories of Orange Park, Florida. We report the reminiscence in full because of Sperry’s involvement in it:

Some of the fun in research comes during discussions, at lunch, at colloquia, and so on. On one such occasion at the Yerkes Laboratories, Lashley and I posed the problem of finding a name for our type of research. Lashley, a zoologist, preferred the name “psychobiology”; I, as a neurosurgeon, opted for “neuropsychology.” Our model for our choices was the term “biochemistry”—the use of chemical techniques to investigate biological problems. Thus Lashley, by this time no longer a behaviorist, wanted to use biological techniques including manipulations of brain function, to investigate psychological processes. I wanted to use behavioral techniques to investigate the organization of brain processes. The small luncheon group (we helped ourselves to the fruit and nuts meant for the laboratory animals) consisted of postdoctoral and graduate students: Bob Blum and Josephine Semmes-Blum-Evarts; Don Hebb; Austin Riesen; Roger Sperry; and Marjory Wade. We all were interested in brain (or at least sensory) function so, for the moment, the term “neuropsychology” won out (Pribram, 2012).

Pribram does not say what term Sperry voted for, but we can guess that he had some preference for psychobiology, though not just to please his boss Lashley.

## Sperry at Caltech

Sperry was recruited by Caltech in 1954 to become the second Hixon professor after the geneticist George Beadle, who won a Nobel prize for Physiology or Medicine for demonstrating the one gene-one protein relation. Frank P. Hixon was a philanthropist businessman who in the 1930s had donated money to Caltech to endow a professorship in the Division of Biology. Norman Horowitz, a professor in the Division of Biology, went to hear a talk by Sperry at a meeting in Massachusetts and wrote the following: “as soon as Roger finished his talk, I knew what I had to do. At Caltech we were searching for the first Hixon Professor of Psychobiology. When I got home, I spoke to the Hixon Search Committee Chairman, Prof. A. van Harreveld, and described Sperry and his work. An invitation to Roger for some lectures followed, and the rest, as they say, is history” (Horowitz, 1994). The history is that Sperry was Hixon Professor of Psychobiology at Caltech for more than 40 years. The Horowitz story suggests that the name psychobiology had already been chosen before recruiting Sperry, but in a recent interview John Allman, Sperry’s successor on Caltech’s Hixon chair, said that Hixon professors have some option in the choice of the title of their professorships (Zierler, 2021). Allman has opted for professor of neurobiology and surmises that Sperry may have been the one to choose psychobiology. If so, Sperry’s choice may have been a homage to the Lashley described by Pribram, or, more romantically, to Herrick’s tradition at Chicago. Fortunately, in this case we have Sperry’s own word about his attitude toward terms such as psychobiology and neuropsychology. In a paper entitled “Psychobiology and vice versa,” Sperry has written the following:

“Psychobiology, like its synonyms biopsychology, psychophysiology, physiological psychology, neuropsychology, neurobiology, behavioral biology, behavioral science, neuroscience etc., is a term that is rather loosely defined and means different things in different places. Regardless of the name of the game, our research strategy is to keep our biological sights trained on the higher functions of the nervous system – the mental, cerebral or psychic activities for which brains are particularly noted. This concern for the higher functions separates psychobiology somewhat from the more broadly defined “neuro” sciences. If some of our projects deal with subjects like “the cytochemical basis of morphogenetic gradients regulating cell adhesivity,” it is not because of any prime interest in molecular phenomena as such, but because some general principle of cerebral integration is at issue. The direct bearing on questions of higher mental function makes the difference” (Sperry, 1968).

In typical sperryan fashion, including the slightly teasing of the long cell biology titles of the sixties, Sperry teaches us that names aren’t the point, that all of them are more or less synonymous, that the real big issue which should always take first place is the search for psyche in the brain. Nevertheless, the emphasis on the “concern for the higher function” may suggest an allusion to Herrick’s psychobiology that we will examine in the last part of the paper.

## Connectionism vs. anticonnectionism

Maverick is the best definition of Roger Sperry as a scientist, experimenter, and thinker (Trevarthen, 1994). In science, a maverick is someone who always works on his own original and independent ideas and refuses to conform with those of others until he has convinced himself of their reliability. Unlike other famous scientists, Sperry did not have an acknowledged maestro or an identified model scientist to be inspired by. In his academic career he had been supervised by zoologist Paul Weiss as his doctoral thesis adviser at the University of Chicago, and then by psychologist Karl Lashley as his post-doctoral sponsor at Harvard and at the Yerkes primate laboratories in Florida. Both Weiss and Lashley were acknowledged leaders in their fields who at the time had seriously challenged the standard picture of central nervous integration deriving from the great histological work of Santiago Ramon y Cajal and the equally great physiological work of Charles Scott Sherrington. The standard sherringtonian picture of central nervous integration envisaged a fundamental correspondence of structural and functional units in neural organization, a functional specialization of different neurons, and a specificity of the synaptic connections between fibers and neurons in functional nervous networks. From different anticonnectionist positions Weiss and Lashley opposed this view by denying the functional specialization of neurons as well as the specificity of synaptic connections. They also attributed to the nervous system an almost unlimited plasticity capable to re-establish orderly function even after pathological or experimental alterations of neuronal structure. Weiss studied the motor innervation of muscles of supernumerary limbs grafted near native limbs in amphibia. Following the section of a motor nerve and its regeneration, innervation of muscles in the grafted and native limbs occurred in an unspecific and diffuse way, but contractions of homologous muscles in native and grafted limbs always occurred in unison. Weiss proposed a resonance theory by which muscles were attuned to, or resonated to, a specific pattern of impulses carried by whatever nerve was in their vicinity. The selectivity of muscle activation during coordinated contraction was therefore due not to specific connections between nerve fibers and muscles, but to a selective muscle activation by a specific impulse code transmitted by diffuse nonselective synaptic connections (Weiss, 1936).

Lashley was a major figure in psychology during the first half of the twentieth century. He argued that the organization of neurons in reflex pathways could at best explain simple movements like scratching, but not higher order behavior involving the cortex. He did not believe that memory engrams could be formed as learning-dependent synaptic changes and claimed that no memory engram could be localized in the brain. He thought that adjacent neurons interacted more by extrasynaptic than synaptic mechanisms and was somewhat attracted by the idea that long fiber connections such as the corpus callosum are not functional links between distant neurons, but skeletal structures keeping the brain together (Lashley, 1930). He established two general principles, the principle of mass action, according to which learning ability is impaired in proportion to the extent of a lesion of the association cortex but not in relation to its locus; and the equipotentiality principle, according to which any intact part of a functionally specialized area can carry out, with or without reduction in efficiency, the functions which are lost by destruction of the whole area (Lashley, 1929). In Sperry’s words, at the end of the 1930s the impact on neurological thinking by Lashley’s principles and other converging lines of anticonnectionist evidence had reduced Sherringtonian connectionism to an example of simplistic and outmoded naivety (Sperry, 1975). Cajal’s neurotropic hypothesis, whereby central nervous organization was thought to come about by an orderly growth of selective neuronal interconnections during embryonic life, had been abandoned in favor of the hypothesis that the developing nervous system started out as an essentially random network, to be shaped into a functionally adaptive system by use and practice, and by elimination of inappropriate connections.

## Sperry’s revolution

Sperry almost singlehandedly reversed this mode of current thinking by showing that synaptic connections are highly selective and behavioral nerve network are congregations of specialized neurons. He found that nerve growth in the brain and spinal centers was anything but diffuse and nonselective; motor nerves and muscles, as well as sensory nerves, were not at all functionally interchangeable after surgical transposition, but instead persistently retained their original functions. Inverted vision of indefinite duration and uncorrectable by experience and training could be produced by surgical eye rotation in fishes and amphibia, even when the optic nerve was cut and allowed to regenerate (Sperry, 1951). To account for these new results Sperry found it necessary to reinstate the old concept of chemotaxis in an even more extreme form, and to postulate a degree of cellular specificity and chemotactic guidance more extensive and refined than that previously imagined even by Ramon y Cajal (Sperry, 1975). As neuroembryologist Viktor Hamburger famously stated, Sperry was the only scientist he knew that had disposed of the cherished theories of his two sponsors already when he was working under them (Bogen, 1999). Sperry proposed a chemoaffinity hypothesis whereby early in development, individual nerve cells acquire and retain individual chemical identification tags, such that lasting functional synaptic connections are established only between neurons that are selectively matched by inherent chemical affinities (Sperry, 1963; Meyer, 1998). This new approach provided a plausible general biological explanation of how behavioral nerve networks grow, assemble, and organize themselves using complex chemical codes under genetic control and with the help of learning mechanisms.

## Sperry vs. Weiss

Weiss lived long enough to see Sperry’s work awarded a Nobel prize in physiology or medicine, and to boast that Sperry had been his best student (Gazzaniga, 2014). But he never fully accepted Sperry’s chemoaffinity hypothesis and more than 20 years after Sperry’s doctoral graduation, the two crossed swords at a meeting of the Neuroscience Research Program on impulse specificity versus connection specificity. Sperry refused to accept a synthesis of his presentation written by Weiss as chairman “because of the long-standing theoretical differences” between their views. He requested and obtained that his presentation be published in its original form along with the Weiss’s resumé (Sperry, 1965c). Sperry recognized that Weiss had been a pioneer in the field of neurogenesis and was grateful to Weiss for introducing him to advanced experimentation in that field, but according to Bernice Grafstein, who knew both well, the “long-standing theoretical difference” was clearly associated, if not identical, with a life-long antipathy (Grafstein, 2001, 2006).

## Sperry vs. Lashley

The relations between Sperry and Lashley was different from that between Sperry and Weiss. During the 5-years of their association as mentor and postdoctoral researcher they coauthored a single paper on the lack of effects of anterior thalamic lesions on olfactory discriminations in rats, a work of marginal interest for their respective convictions about the organization principles of the nervous system (Lashley and Sperry, 1943). Sperry was conscious of Lashley’s profound erudition, brilliant intelligence and strong impact on the thinking of neurology and psychology. Lashley knew that Sperry had exceptional skills as an experimenter and considerably farsighted views about how the brain may work, though he disagreed with Sperry’s firm belief in connectionism. The anticonnectionist Lashley believed that learning and memory are not dependent upon the properties of individual cells but are a function of the total mass of tissue. He also believed in an almost unlimited capacity of the brain to approximate normal function despite extensive structural damage. In one of his thought experiment he speculated that if we could slice off the cerebral cortex and turn it through 180 degrees, getting a random connection of the severed fibers, we might expect to find very little disturbance of behavior (Lashley, 1930). The connectionist Sperry on the contrary believed in the functional specificity of individual neurons and connections, as assumed by the orthodox neural circuit theory. He also made thought experiments and speculated that if topographic projections could be eliminated by random displacement of the nerve cell bodies, at the same time maintaining all the original synaptic connections and conduction-time intervals, little or no disturbance would be expected from the standpoint of orthodox neural circuit theory (Sperry, 1952). Both Lashley and Sperry independently produced evidence against the isomorphic field theory of the Gestalt school, which was acceptable *a priori* to Lashley but not to Sperry (Lashley et al., 1951; Sperry, 1952) One of the strong points of Lashley’s anticonnectionist position was the failure of many animal experiments to show any specific functional deficit from section of the corpus callosum (Glickstein and Berlucchi, 2008), along with the 1940s attempts to treat epilepsy by sectioning the entire corpus callosum in humans, which had produced few if any clear effects, either therapeutic or physiopathological (Akelaitis, 1941). Lashley died in 1958, shortly before Sperry and coworkers were able to provide a definitive solution to the riddle of the corpus callosum in animals and humans alike, but in his last publication he acknowledged that Sperry was on the right track. Sherrington had asserted that binocular fusion is a purely mental phenomenon because the nervous paths from two corresponding points in the two eyes do not reach a common mechanism in the brain (Sherrington, 1951). Lashley disagreed and used interocular transfer of visual discriminations to demonstrate that learning did not depend on the nerve pathway used for learning. In an essay presented at a meeting in 1956, and published in 1958, Lashley admitted that the very recent studies of Sperry on interocular transfer in cats had shown that anatomical connection is essential. “The normal cat has binocular fusion like that of man. What is seen by one eye is recognized by the other. When the optic chiasma is severed, this interocular transfer still occurs, but if the splenium is also severed, reactions which are formed with one eye are not transferred to the other” (Lashley, 1958). In turn, Sperry admitted that some of Lashley’s speculations, though basically untestable or wrong, had inspired some very important works by himself or others. Sperry (1965b) wrote: Karl Lashley surmised that if it were feasible, a surgical rotation through 180 degrees of the cortical brain center for vision would probably not much disturb visual perception. Rotation of the brain center was not feasible, but it was possible to rotate the eyes surgically through 180 degrees in a number of lower vertebrates. This operation was found to produce very profound disturbances of visual perception that were correlated with the geometry of the sensory disarrangement. The animals responded as if everything were to them upside down or reversed from left to right. Contrary to with earlier supposition it appeared that visual perception was very closely tied to the underlying inherited structure of the neural machinery.” And many years later, when holography and interference patterns had become central in the study of vision, in a paper about the importance of metatheories in science and philosophy, Sperry wrote that Karl Lashley …“neither abandoned the reductive approach nor turned to dualism. To account for his path-breaking approach that the engrams of memory are distributed … Lashley proposed his “reduplicated wave interference pattern” hypothesis, which was very much in the reductionistic monist tradition and a 1940s precursor of today’s hologram models of brain function (Sperry, 1992). Like Sperry, another well-known associate of Lashley, Donald Hebb, did not agree with Lashley’s anticonnectionist ideas and wrote a book on a hypothetical, fully connectionistic organization of the brain in mental and behavioral activities (Hebb, 1949). This book has become a classic in the neurosciences and continues to be highly cited to the present day. Modern analyses show that some of Hebb’s ideas can be reconciled with those of Lashley (Nadel and Maurer, 2020).

## Sperry and the mind–body problem

The materialistic-behavioristic thesis that brain function can be described in purely objective terms, without any reference to subjective experience, dissatisfied Sperry already before he started to experiment on split-brain humans. He was aware of the deficiencies of the introspective method, but refused to accept that subjective experiences have no operational value and no place in a working model of the brain (Voneida, 1998). When he and his coworkers found that conscious events were restricted to one hemisphere of patients commissurotomized for treating drug-resistant epilepsy, he started to elaborate a view of inner conscious awareness as a distinct and localized property of the brain, an operational consequence of activity in cerebral systems specialized in the production of select conscious effects. In the years 1964 and 1965, no doubt in recognition of his spectacularly successful split-brain approach to the investigation of mental functions, Sperry was invited as main speaker at three major cultural and scientific events. In June 1964 he gave the 33rd James Arthur lecture on the evolution of the human brain, entitled “Problems outstanding in the evolution of brain function,” at the American Museum of Natural History in New York (Sperry, 1964). In September 1964 he spoke about “Brain bisection and mechanisms of consciousness” at the Study Week on Brain and Conscious Experience, organized in Vatican City in Rome by the Pontifical Academy of Science, to which Sperry was elected in 1978 (Sperry, 1965a). In 1965 he lectured on “Mind, Brain and Humanistic Values” in one of the Monday Lectures on New Views on the Nature of Man, organized by John Platt at Sperry’s alma mater, the University of Chicago (Sperry, 1965b).

At the Vatican meeting, in the primal home of Roman Catholicism, Sperry surprised an audience of leading neuroscientists, including two actual (Lord Adrian and Heymans) and two future Nobel prize winners (Eccles and Granit), by explaining that splitting the brain also splits the mind. Compared with this bold affirmation, less heed was paid to his terse statement that:

… consciousness is an emergent property of certain specialized cerebral circuits in action, that is, circuits that are living and unanesthetized and engaged in a normally alert form of activity … I have often thought that a computer with a sense of pain and pleasure, not to mention color perception, hearing and other feelings in the conscious introspective sense, might well be a much more proficient computer than a similar machine without the conscious properties. For adaptive and complex reactions consciousness may not be necessary, but when it comes to learning that involves memory, conscious centers become a tremendous asset. This reasoning favors the view that consciousness may have a real operational value, that it is more than merely an overtone, a by-product epiphenomenon, or a metaphysical parallel of the objective process (Sperry, 1965a).

In the other two talks of 1964–65, and especially in the Chicago talk, Sperry began to lay down the principles of an emergent theory of mind which was destined to become his overwhelming intellectual occupation for the rest of his life, and eventually to replace all his other research interests responsible for his scientific achievements, including the Nobel prize winning characterization of the different mental properties of the right and left hemispheres of the human brain. Sperry had convinced himself that consciousness is a pattern or configurational force which supervenes on all the other forces as a downward or macro causal factor of brain activity itself, without breaking the basic laws of neurophysiology, chemistry, and physics. He had major difficulties to express this conviction in terms that would make it acceptable to most neuroscientists and especially to professional philosophers and philosophically inclined psychologists. He was attacked on several fronts and spent most of his time and the energy of his clever brain in a stubborn defense of the theory that he regarded as a milestone of the consciousness revolution and his most important contribution to science and culture. In scientific debates he was used to defeat his opponents with the strength of the results of his experiments, but in philosophical debates the education in philosophy that he had received as an undergraduate at Oberlin College from Raymond Stetson, a student of William James, was often insufficient to counter the superior argumentative ability of his adversaries. His clever brain could not think of an experiment to prove the emergence of consciousness in the brain and especially its downward causation of thought and behavior. Hammering the point verbally through the years did not have enough impact to silence the opposition. Followers and admirers of Sperry regret that he did not take the advice of his old humanist friend Carl Rogers at the University of Chicago: we must learn to live with the paradox of two points of view, the behavioristic and the humanistic, that are totally irreconcilable and cannot be both true (Rogers, 1964).

A recent essay by the psychologist and neurophilosopher Alan Baumeister describes the historical and philosophical roots of emergentism in the neurosciences and the role of Sperry, “one of the most prominent and respected historical figures in neuroscience,” in developing a theory of mind and its relationship with matter (Baumeister, 2024). Baumeister’s ruthless analysis, ultimately critical of all forms of emergentism, emphasizes the essentially dogmatic nature and βthe philosophical inconsistency of Sperry’s theory, but admits that some traces of it persist as minor influences on modern works on emergentism. Baumeister writes that in Sperry’s (1964) lecture at the American Museum of Natural History, the assertion that evolution keeps adding new phenomena and new forces regulated by new scientific principles and laws, was not accompanied by any reference to earlier work on emergentism. In a footnote he adds that Sperry must have been aware of such work because in 1957 he reviewed Herrick’s book on the Evolution of Human Nature which discussed consciousness as an emergent feature of evolution. Herrick is cited in Sperry, (1965b) lecture at the University of Chicago, where Sperry says that even some of the tough-minded brain researchers, such as the “outstanding neuroanatomist C. J. Herrick,” would accept that consciousness and mind are emergent properties of the living brain in action (Sperry, 1965b). Sperry’s further claim that these emergent properties have causal power over brain functioning has not fared well either with the tough-minded brain researchers or the philosophers and psychologists. Herrick published his book on the evolution of the human nature in 1956, when also Sperry was a tough-minded brain researcher who however had published four years earlier a splendid, mostly theoretical paper arguing that consciousness has evolved to better control motor output and overt behavior, and that the brain activity in response to a sensory stimulus resembles neither the original stimulus nor the content of sensory experience elicited by it. Rather, it is a premotor or pre-premotor code of the array of the potential motor responses to the stimulus which allows the organism to be prepared to respond by selecting the most appropriate response in the actual situation. This paper of Sperry was carefully studied and appreciated by Herrick who writes in his 1956 book: “A short untechnical paper by Sperry (1952) approaches the mind–body problem from a different standpoint. Our two studies were carried on quite independently with materials and method that had little in common. We differ from each other about some things, and yet our major conclusions are so similar that Sperry’s paper might be used as a condensed summary of my program” (Herrick, 1956, p. 237).” Herrick wrote this when he was 89 and in Sperry’s words.

“probably the most eminent living authority on the apparatus of mind and behavior” … his book is not another elderly scientist’s late fling at philosophy but represents the mature outcome of an active lifelong concern with psychophysical and correlated problems, approached from the vantage point of an intimate and perhaps unequaled working knowledge of brain organization. … the topics range widely from emergent evolution, morals, creativity, through psychomechanics and the indeterminacy principle on down to cerebral structure. Any critical reader is bound to find plenty with which to argue, especially in the first half of the book where Herrick risks judgement in fields rather remote from his specialty. In any case – right wrong or incomplete – Herrick’s concept of the human mind and its relation to brain mechanism deserves serious consideration by anyone concerned with this paramount enigma, whether it be from the standpoint of science religion or philosophy” (Sperry, 1957).

We believe that after Cajal and Sherrington, Herrick was the brain scientist of an earlier generation that Sperry held most in esteem, especially for his belief in the causal efficacy of consciousness and in the power of human values to influence behavior through the brain.

## Epilog

Sperry always encouraged his students to work on major scientific problems, to disregard the more researchable corollary issues and the hairsplitting details, to keep the big picture constantly in sight. As a maverick researcher he always attacked the major unsolved problems by first mistrusting and then by overturning the potential solutions presently favored by the experts of the field and by the majority of the scientific community. When the prevalent belief was that brain connectivity is devoid of functional specificity, virtually random and organized by the environment, he demonstrated that the developing brain possesses a high degree of internal self-organization, prior to and independent of any environmental influences. When it was believed the corpus callosum has no functions because no symptoms were found after its section in experimental animals and neurosurgical patients, he showed that the essential route for the exchange of sensory, motor, and higher-order information between the two brain hemispheres is the corpus callosum. When world famous neurosurgeons proclaimed that the right hemisphere is a subordinate appendage of the left hemisphere and can be removed without causing mental deficits, he showed that in certain non-verbal cognitive ability the right hemisphere is more useful than the left. Then, not yet an elderly scientist, he decided to attack the mind–body problem. Solving it, in William James’s words, would constitute “the scientific achievement before which all past achievements would pale.” Sperry did not accept the established view that reduces everything to physics and chemistry, and ultimately to quantum mechanics or some even more elemental unifying theory. He wanted to show that the world we live in is governed not only by quantum mechanics, but more prominently and much more crucially by the forces of human values. His encounter with philosophy was by no means a fling, but a long-lasting adventure full of tensions, misunderstandings, disappointments and also failures. In our view, if he did not win the argument, he certainly fought a good fight.

## Author contributions

GB: Writing – original draft, Writing – review & editing, Conceptualization. CM: Conceptualization, Funding acquisition, Writing – review & editing.

## References

[ref1] Akelaitis A. J. (1941). Psychobiological studies following section of the corpus callosum a preliminary report. Am. J. Psychiat. 97, 1147–1157. doi: 10.1176/ajp.97.5.1147

[ref2] Bartelmez C. G. (1973). Charles Judson Herrick. Biogr. Mem. Natl. Acad. Sci. 43, 77–108. PMID: 11615505

[ref3] Baumeister A. (2024). The historical and philosophical roots of emergentism in the neurosciences. J. Hist. Neurosci. 33, 73–88. doi: 10.1080/0964704X.2023.2248193, PMID: 37682692

[ref4] Bogen J. E. (1999). Roger Wolcott Sperry (20 august 1913-17 April 1994). Proc. Amer. Philos. Soc. 143, 492–500. 11624452

[ref5] Boring E. G. (1952). “Autobiography” in A history of psychology in autobiography. eds. Boring E. G. Langfeld H. S. Yerkes R. M. (Worcester, mass: Clark University Press)

[ref6] Bruce D. (1985). On the origin of the term “neuropsychology.” Neuropsychologia 23, 813–814. doi: 10.1016/0028-3932(85)90088-0 3908969

[ref7] Dewsbury D. A. (1991). Psychobiology. Am. Psychol. 46, 198–205. doi: 10.1037/0003-066X.46.3.198 2035930

[ref8] Gazzaniga M. S. (2014). Tales from both sides of the brain: A life in neuroscience New York, NY: Ecco/Harper Collins.

[ref9] Glickstein M. Berlucchi G. (2008). Classical disconnection studies of the corpus callosum. Cortex 44, 914–927. doi: 10.1016/j.cortex.2008.04.001, PMID: 18603234

[ref10] Grafstein B. (2001). Bernice Grafstein. Hist. Neurosci. Autobiog. 3, 246–282.

[ref11] Grafstein B. (2006). Roger Sperry: pioneer of neuronal specificity. J. Neurophysiol. 96, 2827–2829. doi: 10.1152/classicessays.00042.2006 17110736

[ref12] Hebb D. (1949). The Organization of Behavior: A neuropsychological theory. New York: John Wiley and Sons.

[ref13] Herrick C. J. (1956). The evolution of human nature Austin and London: University of Texas Press.

[ref14] Horowitz N. (1994). “Roger Sperry comes to Caltech” in A Celebration-August 14, 1994. eds. Roger W. Sperry R. (Pasadena, CA: California Institute of Technology)

[ref15] Kingsland S. E. (1993). A humanistic science: Charles Judson Herrick and the struggle for psychobiology at the University of Chicago. Perspect. Sci. 1, 445–477. doi: 10.1162/posc_a_00443

[ref16] Lashley K. S. (1923). The behavioristic interpretation of consciousness. I. Psychol. Rev. 30, 237–272. doi: 10.1037/h0073839

[ref17] Lashley K. S. (1929). Brain mechanisms and intelligence: A quantitative study of injuries to the brain. Chicago: University of Chicago Press.

[ref18] Lashley K. S. (1930). Basic neural mechanisms in behavior. Psychol. Rev. 37, 1–24. doi: 10.1037/h0074134

[ref19] Lashley K. S. (1958). Cerebral organization and behavior. Res. Pub. Ass. Res. Nerv. Ment. Dis. 36, 1–18. PMID: 13527780

[ref20] Lashley K. S. Chow K. L. Semmes J. (1951). An examination of the electrical field theory of cerebral integration. Psychol. Rev. 58, 123–136. 14834295 10.1037/h0056603

[ref21] Lashley K. S. Sperry R. W. (1943). Olfactory discrimination after destruction of the anterior thalamic nuclei. Am. J. Phys. 139, 446–450.

[ref22] Meyer R. L. (1998). Roger Sperry and his chemoaffinity hypothesis. Neuropsychologia 36, 957–980. doi: 10.1016/S0028-3932(98)00052-9, PMID: 9845045

[ref23] Nadel L. Maurer A. P. (2020). Recalling Lashley and reconsolidating Hebb. Hippocampus 30, 776–793. doi: 10.1002/hipo.23027, PMID: 30216593 PMC6417981

[ref24] Orbach J. (1982). Neuropsychology after Lashley: Fifty years since the publication of brain mechanisms and intelligence. New York: Lawrence Erlbaum Associates.

[ref25] Pribram K. H. (2012). “Autobiography in anecdote. The founding of experimental neuropsychology” in Pathways to prominence in neuropsychology: reflections of twentieth-century pioneers. eds. Stringer A. Cooley E. L. Christensen A.-L. (New York: Psychology Press)

[ref26] Rogers C. R. (1964). Freedom and commitment. Humanist 29, 37–40.

[ref27] Sherrington C. S. (1951). Man on his nature Cambridge, U.K.: Cambridge University Press.

[ref28] Sperry R. W. (1951). Regulative factors in the orderly growth of neural circuits. Growth Symp. 10, 63–87. 13151458

[ref29] Sperry R. W. (1952). Neurology and the mind-brain problem. Amer. Sci. 40, 291–312.

[ref30] Sperry R. W. (1957). Review of C. Judson Herrick, the evolution of human nature. Eng. Sci. 20, 6–10.

[ref31] Sperry R. W. (1963). Chemoaffinity in the orderly growth of nerve fiber patterns and connections. Proc. Natl. Acad. Sci. U.S.A. 50, 703–710. doi: 10.1073/pnas.50.4.703, PMID: 14077501 PMC221249

[ref32] Sperry R. W. (1964). Problems outstanding in the evolution of brain function. New York: American Museum of Natural History.

[ref33] Sperry R. W. (1965a). Brain bisection and mechanisms of consciousness. Pontificiae Academiae Scientiarum Scripta Varia 50, 441–468.

[ref34] Sperry R. W. (1965b). “Mind, brain and humanist values” in New views of the nature of man. ed. Platt J. R. (Chicago: University of Chicago Press)

[ref35] Sperry R. W. (1965c). Selective communication in nerve nets: impulse specificity vs. connection specificity. Neurosci. Res. Prog. Bull. 3, 37–43.

[ref36] Sperry R. W. (1968). Psychobiology and vice versa. Eng. Sci. California Institute of Technology 32, 53–61.

[ref37] Sperry R. W. (1975) In search of psyche. In: Worden F. G., 3. Swazey P. Adelman G. (Eds.), The neurosciences: Paths of discovery, Cambridge: MIT Press.

[ref38] Sperry R. W. (1992). Paradigms of belief, theory and metatheory. Zygon 27, 245–259. doi: 10.1111/j.1467-9744.1992.tb01065.x

[ref39] Trevarthen C. (1994). Sperry Roger W. (1913-1994). Trends Neurosci. 17, 402–404. doi: 10.1016/0166-2236(94)90012-4 7530876

[ref40] Voneida T. J. (1998). Sperry’s concept of mind as an emergent property of brain function and its implications for the future of humankind. Neuropsychologia 36, 1077–1082. doi: 10.1016/S0028-3932(98)00061-X, PMID: 9845054

[ref41] Weidman N. M. (1999). Constructing scientific psychology: Karl Lashley’s mind-brain debates Cambridge, U.K.: Cambridge University Press.

[ref42] Weiss P. (1936). Selectivity controlling the central-peripheral relations in the nervous system. Biol. Rev. 11, 494–531. doi: 10.1111/j.1469-185X.1936.tb00919.x

[ref43] Zierler D. (2021). John M. Allman, frank P. Hixon professor of neurobiology. The Caltech Heritage Project. Available at: https://heritageproject.caltech.edu/interviews/john-allman

